# Campath, calcineurin inhibitor reduction and chronic allograft nephropathy (3C) study: background, rationale, and study protocol

**DOI:** 10.1186/2047-1440-2-7

**Published:** 2013-05-06

**Authors:** Richard Haynes, Colin Baigent, Paul Harden, Martin Landray, Murat Akyol, Argiris Asderakis, Alex Baxter, Sunil Bhandari, Paramit Chowdhury, Marc Clancy, Jonathan Emberson, Paul Gibbs, Abdul Hammad, Will Herrington, Kathy Jayne, Gareth Jones, Nithya Krishnan, Michael Lay, David Lewis, Iain Macdougall, Chidambaram Nathan, James Neuberger, Chas Newstead, Ravi Pararajasingam, Carmelo Puliatti, Keith Rigg, Peter Rowe, Adnan Sharif, Neil Sheerin, Sanjay Sinha, Chris Watson, Peter Friend

**Affiliations:** 1 Clinical Trial Service Unit & Epidemiological Studies Unit, Richard Doll Building, Old Road Campus, Roosevelt Drive, Headington Oxford OX3 7LF, UK; 2 Oxford Kidney Unit, Churchill Hospital, Headington, Oxford OX3 7LJ, UK; 3 Royal Infirmary of Edinburgh, Little France Crescent, Edinburgh EH16 4SA, UK; 4 University Hospital of Wales, Heath Park, Cardiff CF4 4XW, UK; 5 Hull Royal Infirmary, Anlaby Road, Hull HU3 2JZ, UK; 6 Guy’s Hospital, St Thomas Street, London SE1 9RT, UK; 7 Western Infirmary, Dumbarton Road, Glasgow G11 6NT, UK; 8 Queen Alexandra Hospital, Cosham, Portsmouth PO6 3LY, UK; 9 Royal Liverpool University Hospital, Prescot Street, Liverpool L7 8XN, UK; 10 Royal Free Hospital, Pond Street, London NW3 2QG, UK; 11 University Hospitals Coventry and Warwickshire NHS Trust, Clifford Bridge Road, Coventry, West Midlands CV2 2DX, UK; 12 King’s College Hospital, Denmark Hill, London SE5 9RS, UK; 13 Northern General Hospital, Herries Road, Sheffield S5 7HU, UK; 14 NHS Blood and Transplant, Stoke Gifford, Bristol BS34 8RR, UK; 15 St James’s University Hospital, Beckett Street, Leeds LS9 7TF, UK; 16 Manchester Royal Infirmary, Oxford, Manchester M13 9WL, UK; 17 Royal London Hospital, Whitechapel, London E1 1BB, UK; 18 City Hospital, Hucknall Road, Nottingham NG5 1PB, UK; 19 Derriford Hospital, Derriford Road, Plymouth PL6 8DH, UK; 20 Queen Elizabeth Hospital, Edgbaston, Birmingham, West Midlands B15 2TH, UK; 21 Freeman Hospital, High Heaton, Newcastle-upon-Tyne NE7 7DN, UK; 22 Oxford Transplant Centre, Churchill Hospital, Headington, Oxford OX3 7LJ, UK; 23 Addenbrooke’s Hospital, Cambridge CB\2 2QQ, UK; 24 Churchill Hospital, Headington, Oxford OX3 7LJ, UK

**Keywords:** Kidney transplantation, Alemtuzumab, Campath, Sirolimus, Randomized controlled trial, Basiliximab, Tacrolimus

## Abstract

**Background:**

Kidney transplantation is the best treatment for patients with end-stage renal failure, but uncertainty remains about the best immunosuppression strategy. Long-term graft survival has not improved substantially, and one possible explanation is calcineurin inhibitor (CNI) nephrotoxicity. CNI exposure could be minimized by using more potent induction therapy or alternative maintenance therapy to remove CNIs completely. However, the safety and efficacy of such strategies are unknown.

**Methods/Design:**

The Campath, Calcineurin inhibitor reduction and Chronic allograft nephropathy (3C) Study is a multicentre, open-label, randomized controlled trial with 852 participants which is addressing two important questions in kidney transplantation. The first question is whether a Campath (alemtuzumab)-based induction therapy strategy is superior to basiliximab-based therapy, and the second is whether, from 6 months after transplantation, a sirolimus-based maintenance therapy strategy is superior to tacrolimus-based therapy. Recruitment is complete, and follow-up will continue for around 5 years post-transplant. The primary endpoint for the induction therapy comparison is biopsy-proven acute rejection by 6 months, and the primary endpoint for the maintenance therapy comparison is change in estimated glomerular filtration rate from baseline to 2 years after transplantation. The study is sponsored by the University of Oxford and endorsed by the British Transplantation Society, and 18 centers for adult kidney transplant are participating.

**Discussion:**

Late graft failure is a major issue for kidney-transplant recipients. If our hypothesis that minimizing CNI exposure with Campath-based induction therapy and/or an elective conversion to sirolimus-based maintenance therapy can improve long-term graft function and survival is correct, then patients should experience better graft function for longer. A positive outcome could change clinical practice in kidney transplantation.

**Trial registration:**

ClinicalTrials.gov, NCT01120028 and ISRCTN88894088

## Background

Kidney transplantation is well established as the best treatment for patients with end-stage renal failure [1]. Despite significant advances in short-term graft survival over the past two decades, these have not been matched by improved long-term graft survival [2]. Long-term graft survival has many implications both for individual patients (who generally enjoy a better quality of life than when on dialysis) and for healthcare providers (after the initial cost surrounding the operation, the cost of maintaining a graft is less than that of dialysis). The 1-year graft survival rates are now more than 90%, so there is considerable interest in strategies that can maximize the life span of renal transplants.

There are many potential causes of late graft failure, with the most common being interstitial fibrosis/tubular atrophy (IF/TA) [3]. IF/TA is believed to be the end result of various types of graft damage, including preservation damage, rejection, calcineurin inhibitor (CNI) toxicity, hypertensive vascular disease, and viral infection. Functional studies significantly underestimate the incidence of histological graft injury, with one study showing that 94% of grafts had histological evidence of IF/TA at one year [4]. This same study concluded that much of the chronic damage is due to CNI toxicity, even though the levels of these drugs in the study had been maintained within the target range. For this reason, many recent studies have focused on reducing exposure to CNIs, and these have generally shown that this strategy produces better medium-term outcomes (for example, graft function at 1 year) [5]. There are two potential strategies to minimize CNI exposure: more potent induction therapy could be used safely to allow dose reduction or avoidance of CNIs, or CNIs could be replaced by a different class of immunosuppressant that is less likely to damage the kidney.

### Campath is a potent induction agent

Campath (alemtuzumab) is a humanized monoclonal antibody directed against CD52, and causes depletion of lymphocytes. It was first studied in kidney transplantation as a potential treatment for acute rejection. In a small non-randomized pilot study of 12 patients, it appeared to be effective, but was associated with severe infective episodes [6]. The dosage was revised from seven daily doses of 10 mg each to five daily doses of 6 mg each, and no further severe infections occurred in the five patients who received the less potent regimen. It has since been used as induction therapy in many centers; over 1,500 transplants in the USA received Campath induction during the 2-year period of 2003 to 2004 [7].

Until recently there have been very few data on the safety and efficacy of Campath from randomized controlled trials. A systematic review of Campath as induction therapy identified five studies comparing Campath with interleukin-2 receptor antagonist induction, and identified a significant reduction in the risk of acute rejection (relative risk (RR) = 0.54; 95% confidence interval (CI) 0.37 to 0.79), but no effect on short-term graft survival (RR = 1.06; 95% CI 0.64 to 1.78) [8]. However, there were only 659 patients in total included in these trials, so substantial uncertainty remains over the efficacy and safety of Campath. The largest trial to date was the INTAC (Induction with TACrolimus) trial, which compared Campath with basiliximab in 335 low-risk patients (defined by non-black ethnicity, first transplant, and panel reactive antibody (PRA) level <20) [9]. At 1 year, the rate of biopsy-proven acute rejection was 3% in participants allocated Campath versus 22% in those allocated basiliximab (*P*<0.001). There was a small excess risk of serious infection in participants allocated Campath (57 versus 38 events, *P* = 0.02), but not of any infection (129 versus 123 events, *P* = 0.17). However, the INTAC trial did not attempt to spare CNI after Campath: all participants received the same maintenance immunosuppression of tacrolimus (target trough concentration 7 to 14 ng/ml for 6 months then reducing to 4 to 12 ng/ml), mycophenolate mofetil (2 g/day) and corticosteroids (1 g or less prednisolone equivalent during the first 5 days), so substantial uncertainty remains over the safety and efficacy of Campath as part of a CNI-minimization strategy.

### Sirolimus as a potential replacement for calcineurin inhibitors

Sirolimus is a macrocyclic lactone, and has a different mechanism of action to CNIs. It blocks the mammalian target of rapamycin pathway, thus inhibiting cellular proliferation. Sirolimus has been used in a variety of strategies for kidney transplantation. It was initially used *de novo* (in conjunction with ciclosporin) but is not as effective as CNIs during the high-risk post-operative period, and the doses required to prevent rejection (including a high loading dose) were associated with unacceptable adverse effects [10]. Trials of late conversion to sirolimus failed to show any benefit [11], but more recently trials of early (that is, within 3 to 6 months post-transplant) conversion to sirolimus have shown potential. The CONCEPT study randomized 192 kidney-transplant recipients to remain on a ciclosporin-based regimen or to switch to a sirolimus-based regimen at 3 months after transplantation. The patients allocated to sirolimus had better graft function at 1 year compared with the ciclosporin-allocated group (Cockcroft-Gault glomerular filtration rate 68.9 versus 64.4 ml/min, *P*=0.017) with no significant excess of acute rejection [12]. A similarly designed study using everolimus (initiated at 4.5 months post-transplant) in 300 patients also showed a highly significant increase in eGFR at 1 year after transplant (eGFR 71.8 versus 61.9 ml/min/1.73m^2^, *P*<0.0001) [13]. The benefits appear to be durable in the medium term [14], but whether these translate into differences in clinical outcomes such as graft failure remains uncertain.

### Campath and sirolimus in tolerance

The combination of Campath and sirolimus may enable exposure to CNIs to be reduced or eliminated. This could be favorable because CNIs are nephrotoxic and may interfere with tolerogenesis [15]. It is known that ischemia-reperfusion injury occurring during organ implantation enhances the activation of the immune system [16]. Depleting induction agents profoundly reduce the number of circulating lymphocytes capable of mounting an immune response during this period. It has been suggested that by the time the peripheral lymphocytes return, the graft may have recovered from the injury, and will therefore be immunologically quiescent [17]. Studies examining the use of Campath followed by either tacrolimus [18] or sirolimus [19] monotherapy have had encouraging results, consistent with (but not yet proving) the concept of donor-specific hyporesponsiveness suggested by Calne when he proposed the term *prope* (almost) tolerance [20].

Sirolimus is also potentially tolerogenic; it increases the number of CD4+ cells with a regulatory phenotype (Treg cells) [21]. Treg cells dampen the effector response to antigenic challenge and are a crucial element of peripheral tolerance. Furthermore, in addition to its effects on tolerance-promoting Treg cells, sirolimus facilitates the deletion of effector alloreactive T cells [22]. In combination, Campath and sirolimus have been shown to induce donor-specific hyporesponsiveness, as assessed by *in vitro* tests [23]. This is obviously encouraging, and merits further investigation.

Based on these observations, we have designed a clinical trial with the purpose of testing whether Campath and/or sirolimus can improve long-term outcomes after kidney transplantation.

## Methods/Design

### Basic protocol overview

The 3C Study is an open-label, randomized multi-center trial comparing 1) Campath-based and basiliximab-based induction therapy strategies; and 2) from about 6 months after transplantation, sirolimus-based and tacrolimus-based maintenance therapy strategies. The study is planned to take about 7 years, with recruitment of the 852 participants taking 2 years, followed by a 5-year follow-up period.

**Figure 1:**
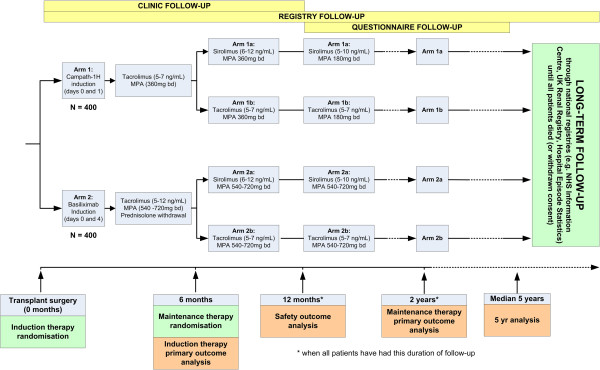
Flowchart showing study treatments and randomizations, including proposed analyses.

Participants will be randomly allocated to receive either Campath-based or basiliximab-based induction therapy before transplantation. All participants will then receive 6 months of tacrolimus-based maintenance therapy before being randomized again (if they remain willing and eligible) to either sirolimus-based or tacrolimus-based long-term maintenance therapy (Figure 1).

### Inclusion criteria

The 3C Study includes all patients eligible for kidney transplantation, including those receiving kidneys from deceased donors (brain or circulatory death) and living donors, as well as highly sensitized (defined as calculated reaction frequency >85%) and previously transplanted recipients. The specific inclusion criteria are recipients of a kidney-only transplant aged over 18 years.

### Exclusion criteria

Patients will be excluded if they: are pregnant; are receiving multi-organ transplants (including kidney-pancreas transplants); have previously been treated with Campath; have active infection including HIV or viral hepatitis; have a history of anaphylaxis to humanized monoclonal antibodies; have a history of malignancy (except non-melanoma skin cancer) that was diagnosed or recurred in the previous 5 years; have lost a previous kidney transplant within 6 months not due to technical reasons; or have a medical history that might limit their ability to take trial treatments for the duration of the study.

Participants are eligible for the maintenance therapy randomization (sirolimus or tacrolimus) if: 1) at about 6 months after transplantation, their urine protein excretion rate is below 800 mg/day (urine protein:creatinine ratio <80 mg/mmol or albumin:creatinine ratio <50 mg/mmol); and 2) they have not had biopsy-proven acute rejection (Banff grade >1) in the previous 30 days.

### Study objectives

The primary aims of the 3C Study are to assess the differences in 1) biopsy-proven acute rejection among participants allocated Campath-based versus basiliximab-based induction therapy (assessed at 6 months); and 2) graft function among all those allocated tacrolimus-based versus sirolimus-based maintenance therapy (assessed at 2 years after transplantation).

Secondary aims include assessments of the study treatments (Campath versus basiliximab, and tacrolimus versus sirolimus) on: 1) graft-related outcomes (including graft survival, rates of biopsy-proven rejection); and 2) safety outcomes (including infection [particularly opportunistic infections], malignancy and overall survival).

### Randomization and treatment scheme

Participants will be randomized by an internet-based system before transplantation. After the participant is registered on the internet-based system, they are assigned a unique identifier (participant ID), and once randomized, the assigned treatment group is displayed on the screen. Participants assigned Campath-based induction will receive Campath 30 mg (intravenously or subcutaneously) after reperfusion of the transplant (and a further 30 mg 24 hours later if they are ≤60 years old). Before the first dose of Campath, patients will be given 500 mg methylprednisolone and 10 mg chlorphenamine intravenously, but no further steroids will be given. Maintenance oral immunosuppression will consist of mycophenolate sodium (360 mg twice daily) and tacrolimus (starting at 2 mg twice daily from day 3, aiming for target trough concentration 5 to 7 ng/ml). After 12 months, the mycophenolate sodium dose will be reduced to 180 mg twice daily. Participants assigned basiliximab-based induction will receive 20 mg basiliximab intravenously pre-operatively and on day 4, oral mycophenolate sodium (540 to 720 mg twice daily) and oral tacrolimus (0.05 to 0.10 mg/kg twice daily, aiming for target trough concentration 5 to 12 ng/ml). Patients will be given 500 mg methylprednisolone intravenously pre-reperfusion and maintenance oral corticosteroids, starting at 15 to 20 mg prednisolone, to be reduced or withdrawn completely in accordance with local practice (avoiding complete withdrawal 5 to 7 months post-transplantation; that is, around the time of the maintenance therapy randomization).

Participants can enter the maintenance-therapy randomization between 5 to 7 months after transplantation, assuming no exclusion criteria apply. Participants allocated tacrolimus-based maintenance therapy will continue their current therapy and the target trough concentration is 5 to 7 ng/ml in all participants. Participants assigned sirolimus-based maintenance therapy will stop tacrolimus after an evening dose and start sirolimus the next morning at 3 mg daily (unless they weigh <60 kg, when 2 mg daily will be used). Target trough concentration is 6 to 12 ng/ml for the first 6 months, then reducing to 5 to 10 ng/ml. Advice will be given on mouth, care and a short course of low-dose prednisolone can be used to cover the conversion period if the local investigator considers it necessary. Mycophenolic acid levels are not routinely monitored in the UK, so are not specified in this protocol.

Both randomizations use a minimization algorithm that ensures balance for recipient age, ethnicity, type of transplant, human leukocyte antigen mismatch, sensitization status (and for maintenance randomization, allocated induction therapy).

Other treatments (including cytomegalovirus and *Pneumocystis* prophylaxis) will be left to the discretion of the local investigator. A summary of the treatment scheme and flow of participants through the trial is shown in Figure 1.

### Follow-up and documentation

During the first year after transplantation, all participants will be followed up at discharge after transplantation and at 1, 3, 6, 9, and 12 months after transplantation. Data will be collected on all serious adverse events (which include all episodes of rejection and opportunistic infection for the purposes of this study), current medication (including doses of immunosuppressive drugs), non-serious adverse events considered to be related to one of the study treatments, along with blood pressure and weight, and relevant laboratory values (including serum creatinine, full blood count, lipid profile, and urine protein/albumin to creatinine ratio). Data will also be collected on healthcare usage and quality of life to allow health economic analyses to be conducted.

On a yearly basis, all participants will be sent an annual questionnaire to collect information on serious adverse events, study treatments, healthcare usage, and quality of life. In addition, all participants will be flagged with a number of national registries so that their routinely collected data can be used for long-termfollow-up. These registries include the UK Transplant Registry (which collects data on graft survival and function), Office for National Statistics (which collects data on death), National Health Service (NHS) Information Centre (which collects data on cancer), and the Hospital Episode Statistics registry (which collects data on all hospital admissions).

### Endpoint definition

The primary endpoint of the induction therapy comparison will be biopsy-proven acute rejection during the first 6 months after transplantation. The Banff classification definition (including those of the various subtypes) will be used. The histological appearances of cellular rejection after depleting induction with alemtuzumab are usually typical (despite the profound lymphopenia) [24]. All reports of rejection (including events that may yield a diagnosis of rejection (for example, transplant biopsy) will be adjudicated by trained clinicians, blinded to study treatment allocation, at the coordinating center. The date of the rejection will be the date of the diagnostic biopsy.

The primary endpoint of the maintenance therapy comparison will be change in graft function (estimated using the four-variable Modification of Diet in Renal Disease (MDRD) formula [25]) from 6 months to 2 years after transplantation.

Secondary endpoints for both comparisons will include safety outcomes (including infection and cancer) and long-term outcomes (including graft and patient survival).

### Trial organization

The 3C Study is an investigator-initiated trial. Preliminary investigator meetings were organized by the University of Oxford, which is the sponsor of the study. The trial is funded by grants from the UK National Health Service Blood and Transplant Research and Development fund, Pfizer (Collegeville, PA, USA), and Novartis UK.

### Participating centers

Major kidney transplant centers from the UK (18 sites in total) will be participating in the study. Such a collaboration is required in order to recruit the planned number of kidney-transplant patients needed to provide statistically reliable and clinically meaningful results.

### Drug supply

Study drugs will be purchased by participating hospitals, and the Campath will be relabeled by local hospital pharmacies in accordance with the EU Clinical Trial Directive. The other investigation medicinal products (basiliximab, tacrolimus, and sirolimus) will be exempt from the EU Clinical Trial Directive requirements for labeling and accountability, as they will be used within the terms of the marketing authorization. All treatments will be used on an open-label basis.

### Monitoring

Before recruitment started, all sites were visited by the sponsor in order to train the relevant staff in the study procedures. Recruitment rates and completeness of follow-up data will be monitored closely by the sponsor. Sites will be monitored during recruitment and follow-up through a combination of on-site visits from the sponsor and central statistical monitoring. An independent data monitoring committee (DMC) has been convened (see below).

### Ethics and safety

The most recently approved version of the protocol is version 5, which was approved by the Nottingham 2 Research Ethics Committee on 28 February 2012. Trust management approval has been granted by each transplant center. The 3C Study complies with the principles of Good Clinical Practice. Written informed consent will be obtained from each participant before randomization, after a discussion with the local investigator or their nominated deputy. Unblinded interim analyses of all relevant data will be reviewed twice a year by the independent DMC, which can advise the study steering committee if the study protocol needs to be amended in any way, or if they recommend early termination of the study.

### Sample size

The sample size for the 3C Study was determined by the maintenance therapy allocation. A meta-analysis of the effect of conversion to sirolimus-based maintenance therapy showed an improvement in eGFR of 6.4 ml/min/1.73m^2^ (95% CI 1.9 to 11) in the group assigned to sirolimus [26]. The trials included in that meta-analysis varied in duration, with most patients followed up for 1 year. If these differences were maintained, it would be reasonable to anticipate a 10 ml/min/1.73m^2^ difference in eGFR 2 years after conversion to sirolimus (that is, median follow-up at least 2.5 years after transplantation). We assumed that the adherence to sirolimus therapy would be around 75% (that is, approximately 25% of patients allocated sirolimus in randomized trials discontinue it [26]), and further it was estimated that about two-thirds of participants would be willing and eligible to be randomized at 6 months after transplantation. Thus, of 800 patients entering the study, about 530 (two-thirds) would be re-randomized at 6 months. This number of participants would provide excellent power (>90%) with α = 0.05 and good power (>80%) with α = 0.01 to detect such a difference (even if adherence to allocated treatment was only 75%). If the self-correlation between baseline eGFR (that is, before re-randomization at 6 months) and eGFR 2 years later is >0.5, then comparing the change from baseline in eGFR will provide even better power for these analyses.

Having 800 patients would also provide good power (90%) with α = 0.05 to detect a halving in the acute rejection rate at 6 months (from 15% to 7.5%), which is the primary comparison in the induction-therapy comparison.

### Statistical evaluation

The primary endpoint of the induction-therapy comparison (biopsy-proven acute rejection occurring before maintenance-therapy randomization or at 6 months post-transplant (whichever occurs first) will be compared using the log-rank test, with average event rate ratios derived using standard methods [27]. Secondary endpoints of the induction-therapy comparison will be analyzed in an exploratory manner, as there is a potential for bias in endpoints that occur after the maintenance-therapy randomization (if inclusion in the maintenance-therapy comparison is not balanced between the two induction-therapy groups).

The statistical analysis of the primary endpoint of the maintenance treatment will depend on the self-correlation of eGFR at baseline (that is, at randomization into the maintenance comparison) and at 2 years. This will be performed by investigators blinded to treatment allocation and, if the self-correlation is >0.5, the analysis will be of the difference in the mean change from baseline between the two groups using analysis of covariance methods, as this will provide greater power for the analysis (analyses of other datasets suggests this is likely, but if the self-correlation is less than 0.5 then the analysis will be of the difference in mean eGFR at 2 years). All analyses will be intention to treat, and will include all randomized participants who are transplanted. The small number of participants who are randomized but not transplanted will be censored at day 0 for the purposes of analysis.

Secondary endpoints for which time-to-event data are available will be compared with the log-rank test, whereas other categorical endpoints will be compared with χ^2^ tests. Continuous variables will be compared with *t*-tests (after logarithmic transformation if necessary for skewed variables). Tertiary endpoints (that is, subgroup analyses of the primary endpoint in different types of participants) will be interpreted cautiously, as the power to detect true differences between subgroups will be limited. Subgroups will be compared by testing for heterogeneity of the treatment effect across subgroups.

## Discussion

Late graft loss (that is, more than 1 year after transplantation) is a major issue for kidney-transplant recipients. Despite improvements in short-term graft survival, long-term graft survival has not improved substantially [2]. The commonest reason for such late graft failure is IF/TA. Data from serial protocol biopsies previously suggested that CNI nephrotoxicity was an important cause of IF/TA [4], although this has been debated more recently [28,29]. Previous CNI minimization studies have not been large enough or of sufficient duration to detect benefits in terms of long-term graft function and survival. Two potential strategies to reduce exposure to CNIs are Campath as a more potent induction therapy, and conversion to a sirolimus-based maintenance regimen. Given the favorable effects of both treatments on markers of tolerance [30], the 3C Study has been designed to test both strategies to investigate whether they could improve both short-term and long-term outcomes.

The 3C Study deliberately includes a wide range of kidney-transplant recipients. as all transplants are vulnerable to the nephrotoxic effects of CNIs, and it is important to ensure that the results will be applicable to as many kidney-transplant recipients as possible. Many previous trials have only included low-risk transplant recipients, and therefore uncertainty remains about the applicability of such treatments to higher-risk recipients. Using broader exclusion criteria and conducting a small number of carefully pre-specified subgroup analyses will provide reliable information about whether any effect of either strategy is modified by certain baseline characteristics. One such key subgroup analysis will be whether there is an interaction between the two randomizations; for example, whether induction therapy based on Campath modifies the treatment effect (possibly by improving compliance) of sirolimus-based maintenance therapy.

The protocol is intended to be pragmatic and easy to implement at local transplant centers. Considerable effort has been made to make the control treatment as similar to current European practice as possible to ensure that centers would be willing to deliver it to participants and to ensure that the results will be relevant to current practice. Prednisolone withdrawal, precise mycophenolate dosing, and infection prophylaxis will be left to the local investigator’s discretion, in order to limit the effects of the study on routine practice and thus facilitate recruitment. 852 participants have been randomized into the study (of whom 355 have been re-randomized into the maintenance comparison at the time of submission). The safety analysis (once all participants have completed the 1-year follow-up) will therefore be conducted in early 2014.

An important and novel aspect of the 3C Study design is linkage with registries. In the UK, data will be routinely collected on patient survival and certified cause of death (by the Office for National Statistics), cancer incidence (by the NHS Information Centre), hospital admission or outpatient evaluation (by the Hospital Episode Statistics registry), and transplant function, rejection and survival (by the UK Transplant registry). Specific approval to flag all 3C Study participants with these registries has been obtained, and these will therefore provide a cost-efficient means of collecting data on all the relevant outcomes for the lifetime of the participant (unless they withdraw consent). It will therefore be possible to investigate the very long-term effects of the study treatments reliably at reasonable cost. For example, graft failure and cancer (with the possible exception of post-transplant lymphoproliferative disorder) will be uncommon during the first few years after transplantation, when most studies cease follow-up. However, the 3C Study will continue follow-up for many more years, and therefore will accrue substantially more events and thus allow more reliable conclusions to be drawn.

## Abbreviations

3C Study: Campath Calcineurin inhibitor reduction and Chronic allograft nephropathy; CNI: Calcineurin inhibitor; eGFR: Estimated glomerular filtration rate; IF/TA: Interstitial fibrosis/tubular atrophy; MDRD: Modification of Diet in Renal Disease; NHS: National Health Service

## Competing interests

The 3C Study has received funding from Pfizer and Novartis and a NHS Blood and Transplant Research and Development grant. PF has received honoraria from Novartis and Pfizer. PG has received honoraria from Roche and Novartis. KR has received honoraria from Astellas. AA, MA, SB, PC, AH, IM, GJ, NK, JN, CNa, CNe, CP, RP, AS, NS, PR, and CW have no conflicts of interest to declare. The Clinical Trial Service Unit has a staff policy of not accepting honoraria or other payments from the pharmaceutical industry, except for the reimbursement of costs to participate in scientific meetings.

## Authors’ contributions

RH contributed to the study design, coordination, approval processes, and acquisition of data, and drafted the manuscript. PF initiated the study concept and design, and reviewed the paper. CB and ML contributed to the study design and coordination. PH, MA, AA, AB, SB, PC, MC, JE, PG, AH, WH, KJ, GJ, NK, ML, DL, IM, CNa, CNe, JN, RP, CP, KR, PR, AS, NS, SS, and CW contributed to the design, acquisition of data, and revision of the manuscript. All authors read and approved the final manuscript.
